# Gene Characterization and Enzymatic Activities Related to Trehalose Metabolism of In Vitro Reared *Trichogramma dendrolimi* Matsumura (Hymenoptera: Trichogrammatidae) under Sustained Cold Stress

**DOI:** 10.3390/insects11110767

**Published:** 2020-11-07

**Authors:** Xin Lü, Shi-chou Han, Zhi-gang Li, Li-ying Li, Jun Li

**Affiliations:** Guangdong Key Laboratory of Animal Conservation and Resource Utilization, Guangdong Public Laboratory of Wild Animal Conservation and Utilization, Institute of Zoology, Guangdong Academy of Sciences, 105 Xingang Road West, Guangzhou 510260, China; hansc@giabr.gd.cn (S.-c.H.); leegdei@163.com (Z.-g.L.); liyingl32@163.com (L.-y.L.)

**Keywords:** trehalase, trehalose metabolism, in vitro rearing, cold stress, *Trichogramma*

## Abstract

**Simple Summary:**

Trehalose is a non-reducing disaccharide that presents in a wide variety of organisms, where it serves as an energy source or stress protectant. Trehalose is the most characteristic sugar of insect hemolymph and plays a crucial role in the regulation of insect growth and development. *Trichogramma* species are economically important egg parasitoids, which are being mass-produced for biological control programs worldwide. Many *Trichogramma* species could be mass reared on artificial mediums (not insect eggs), in which components contain insect hemolymph and trehalose. These in vitro-reared parasitoid wasps were strongly affected by cold storage, but prepupae could be successfully stored at 13 °C for up to 4 weeks. The aims of the present study were to determine the role of trehalose and the relationship between trehalose and egg parasitoid stress resistance. Our study revealed that (1) trehalose regulated the growth under sustained cold stress; (2) prepupal stage is a critical developmental period and 13 °C is the cold tolerance threshold temperature; (3) in vitro reared *Trichogramma dendrolimi* could be reared at temperatures of 16 °C, 20 °C, and 23 °C to reduce rearing costs. This finding identifies a low cost, prolonged development rearing method for *T. dendrolimi*, which will facilitate improved mass rearing methods for biocontrol.

**Abstract:**

*Trichogramma* spp. is an important egg parasitoid wasp for biocontrol of agriculture and forestry insect pests. Trehalose serves as an energy source or stress protectant for insects. To study the potential role of trehalose in cold resistance on an egg parasitoid, cDNA for trehalose-6-phosphate synthase (TPS) and soluble trehalase (TRE) from *Trichogramma dendrolimi* were cloned and characterized. Gene expressions and enzyme activities of *TdTPS* and *TdTRE* were determined in larvae, prepupae, pupae, and adults at sustained low temperatures, 13 °C and 16 °C. *TdTPS* and *TdTRE* expressions had similar patterns with higher levels in prepupae at 13 °C and 16 °C. *TdTPS* enzyme activities increased with a decrease of temperature, and *TdTRE* activity in prepupae decreased sharply at these two low temperatures. In vitro reared *T. dendrolimi* could complete entire development above 13 °C, and the development period was prolonged without cold injury. Results indicated trehalose might regulate growth and the metabolic process of cold tolerance. Moreover, 13 °C is the cold tolerance threshold temperature and the prepupal stage is a critical developmental period for in vitro reared *T. dendrolimi*. These findings identify a low cost, prolonged development rearing method, and the cold tolerance for *T. dendrolimi*, which will facilitate improved mass rearing methods for biocontrol.

## 1. Introduction

Sugars are used for energy production and are stored as glycogen in the body fat, or as trehalose in the hemolymph [1,2,3]. *Trichogramma* are egg parasitoid wasps that obtain diverse nutrients, including sugars, from their host eggs during development. *Trichogramma dendrolimi* Matsumura is an important biological control agent that has been mass produced on eggs of *Corcyra cephalonica* (Stainton) and *Antheraea pernyi* (Guérin-Méneville) for biological control programs in China [4,5]. Artificial host eggs are now used to mass produce *T. dendrolimi* [6,7]. Lü et al. [8,9,10] developed an artificial medium containing trehalose for the continuous rearing of *T. dendrolimi* and revealed that trehalose was an essential ingredient of the artificial media. Biochemical characteristics, including trehalose content and trehalase activity in *T. dendrolimi,* continuously reared on artificial medium (in vitro) versus those reared on *A. pernyi* eggs (in vivo), were also studied [11]. The quality of in vitro reared *T. dendrolimi* was strongly affected by cold storage, but prepupae could be successfully stored at 13 °C for up to 4 weeks [12].

The developmental temperature threshold can vary, not only among insects, but also among populations [13,14]. *Trichogramma* spp. show different reactions to low temperatures when reared on different hosts or media [12,13]. For *T. dendrolimi*, the developmental threshold temperature was different among geographical populations and hosts: 10.34 °C for south population reared on *Philosamia cynthia ricini* [13]; 10.1 °C for the south population reared on *C. cephalonica* [14]; 5.34 °C/5.1 °C, 5.82 °C/5.42 °C, 11.03 °C/14.83 °C, and 12.37 °C/11.58 °C for the north population (egg, larva, prepupa and pupa) reared on *A. pernyi* [15,16]. The north population reared on *A. pernyi* was unable to complete the entire development (stopped developing at prepupal stage) at 10 °C, but was able to complete the entire development above 15 °C [15]. Compared with the in vivo (on *A. pernyi*) reared *Trichogramma*, in vitro (on artificial medium) reared *Trichogramma* of the south population was more affected by cold storage [12]. All of these experimental populations have been lab adapted. Limited information is available about inducing cold stress on the lab adapted strain of this particular insect species, and molecular mechanisms of trehalose metabolism, or the relationship between trehalose and egg parasitoid stress resistance [17,18,19,20,21]. Based on the facile sampling and reproducibility of the condition of in vitro reared *T. dendrolimi*, trehalose metabolism related enzymes were explored by investigating their changes in gene expression and corresponding enzyme activities to study the effect of cold stress conditions on egg parasitoids.

Trehalose is involved in the regulation of parasitoid growth and development. The disaccharide sugar trehalose serves as an energy source or stress protectant for parasitoids [22,23]. It promotes longevity, fecundity [24,25], and cold tolerance [18,26,27], and provides^,^ energy needed to search for, and parasitize, hosts [28]. Trehalose is synthesized by trehalose-6-phosphate synthase (TPS, EC 2.4.1.15) and trehalose-6-phosphate phosphatase (TPP, EC 3.1.3.12) in the body fat, and is hydrolyzed by trehalase (TRE, EC 3.2.1.28) to yield two glucose molecules in the hemolymph [19,20,29,30,31]. The activity of these three enzymes also affects insect physiology and development. Trehalose and trehalase are closely associated with growth and development throughout insect life cycles [2,23]. However, we should notice the adult yields and quality of in vitro rearing some parasitoids (e.g., the tachinid *Exorista larvarum*) did not drop when the artificial medium without insect material, which trehalose has been replaced with sucrose and sucrose, was even deleted, without drops in adult yields [32,33].

TPS and TPP genes have two functional conserved domains similar to yeast genes and are homologs of yeast Tps1 (Ots A) and Tps2 (Ots B), respectively [34]. In insects, TPS is a fused gene [35] and two exons are involved in encoding trehalose synthetase [36]. Trehalase catalyses, the irreversible hydrolysis of trehalose to glucose, which is the only known pathway of trehalose utilization [2]. TRE is essential for energy metabolism and is important in insect growth and molting [37]. Trehalose may function as a cryoprotectant to stabilize proteins at low temperatures [2,18], and may also protect insects from external interference, assist in successful completion of metamorphosis, and aid survival in adverse environments [38]. In addition, trehalose is important in the regulation of insect growth and development and serves as an energy source and stress protectant [19,38,39,40,41].

TPS has been cloned, characterized, and purified from many insect species. It was first cloned from *Drosophila melanogaster* [42]. TRE has been identified in insect species, such as *Rhodnius prolixus*, *Nilaparvata lugens*, *Spodoptera exigua*, *Omphisa fuscidentalis,* and *Harmonia axyridis* [19,30,43,44,45,46]. Although TPS and TRE are important key enzymes for many insects, in parasitoid wasps, only the trehalase cDNA from *Pimpla hypochondriaca* has been cloned [47]. No trehalose metabolism genes in *Trichogramma*, or even in any egg parasitoids, have been characterized. To understand the role of trehalose in the cold resistance of in vitro reared *T. dendrolimi*, two genes (*TdTPS* and *TdTRE*) were identified, and cloned the full-length cDNA of *T. dendrolimi* reared on artificial medium using transcriptome data from *T. dendrolimi* reared on eggs of *A. pernyi*. The changes in gene expression and corresponding enzyme activities in four developmental stages (larva, prepupa, pupa, and adult) as each stage developed at low temperatures were also recorded. The trehalose metabolism in the life cycle of *T. dendrolimi* was systematically investigated in relation to cold hardiness.

## 2. Materials and Methods

### 2.1. Insects

*Trichogramma dendrolimi* were provided by Engineering Research Center of Natural Enemies, Institute of Biological Control, Jilin Agricultural University, Changchun, China. In the laboratory, *T. dendrolimi* stock cultures were reared on eggs of *A. pernyi* as a factitious host. Rearing conditions were 27 °C ± 1, 75% ± 5 relative humidity (RH) and a 16:8 h (L:D) photoperiod.

### 2.2. Preparation of Artificial Medium and Insect Rearing

The artificial medium used in this study was the modified artificial medium developed by Lü et al. [9]. It comprised 3 mL of the pupal hemolymph of *A. pernyi*, 2.5 mL egg yolk, 1 mL 10% malted milk solution, 1 mL Neisenheimer’s salt solution, 0.1 g trehalose (Sigma, St. Louis, MO, USA), and 1.5 mL sterile water. The preparation of artificial egg cards was done as described by Lü et al. [11].

Artificial egg cards were placed in a plastic tray (20 cm × 10 cm × 3 cm) for 24 h exposure to *T. dendrolimi* adults of the same batch. Parasitoids of both sexes were released in the trays using a 6:1 ratio of parasitoids to artificial eggs. Sex ratios were approximately 8:1 (female:male) in all three replicates (one tray corresponded to one replicate). Trays were placed in climatic incubators (Yamato, Tokyo, Japan) set at 27 °C ± 1, 75% ± 5 RH, and a 16:8 h (L:D) photoperiod. After 24 h of exposure, the wasps were removed, and the egg cards were transferred to the temperature treatments [9].

### 2.3. Experimental Set-Up, Sample Collection, and Biological Parameters Assessment

In a pre-experiment, in vitro reared *T. dendrolimi* were unable to complete the entire development (from egg to adult) at 10 °C, and stopped developing at prepupal or pupal stages. The optimum storage condition for these parasitoid wasps are prepupae that can be stored at 13 °C for up to 4 weeks without affecting reproductive quality [12]. The present experiment had two factors: temperature (13 °C ± 1 (optimum storage temperature), 16 °C ± 1 (above the optimum storage temperature) and 27 °C ± 1 (optimum development temperature)), and developmental stage (larva, prepupa, pupa, and adult). In a preliminary experiment, based on the transparency of the egg cards, they can be easily monitored daily for parasitoid development using a binocular microscope. *T. dendrolimi* developing to new larvae, prepupae, pupae, or adults were collected on ice and maintained at −80 °C for gene expression and biochemical assessments.

In the follow-up rearing experiment, to investigate the developmental quality of *T. dendrolimi* reared on artificial medium at different temperatures (27 °C ± 1, 23 °C ± 1, 20 °C ± 1, 16 °C ± 1, and 13 °C ± 1), the developmental durations (from oviposition to adult emergence) of the eggs, larvae, prepupae, and pupae, number of male adults and total adults observed per egg card were examined. Pupation rate, adult emergence rate (based on pupal numbers), numbers of normal adults produced (i.e., adults not having an enlarged abdomen and/or unexpanded wings) and the adult sex ratio (female proportion) was calculated as follows:

– Pupation rate (%) = (number of pupae/total number of larvae observed per egg card) × 100.
– Emergence rate (%) = (number of adults/(number of adults + dead pupae + dead larvae)) × 100.
– Number of normal adults = total number of normal adults observed to emerge from three replicates (egg cards)/3.
– Female proportion (%) = (total number of adults observed − number of male adults observed)/total number of adults observed × 100.

### 2.4. Total RNA Extraction and Cloning of the Full-Length cDNA

Total RNA was extracted from *T. dendrolimi* adults using a TransZol Up Plus RNA Kit (TransGen, Beijing, China). The RNA integrity and concentration were checked by agarose gel electrophoresis and spectrophotometry (NanoDrop2000, Wilmington, DE, USA), respectively. The fragments of *TdTPS* and *TdTRE* were obtained by transcriptome sequencing of *T. dendrolimi* (Hiseq 2000, Illumina, Beijing, China). Full-length sequences of TPS and TRE were obtained by 5′ and 3′ rapid amplification of cDNA ends (RACE) with the SMART^TM^ RACE Kit (TaKaRa, Tokyo, Japan), according to manufacturer instructions. 5′ and 3′ RACE were performed by nested PCR including Universal Primer Mix (UPM) and Nested Universal Primer (NUP) along with gene-specific primers (GSP) (Table 1). The PCR conditions were as follows: initial at 94 °C for 5 min, 32 cycles of 30 s at 94 °C, 30 s at 60 °C, 2 min at 72 °C, and a final extension at 72 °C for 10 min. The products were examined by agarose gel electrophoresis, purified using a SanPrep Column DNA Gel Extraction Kit (Sangon, Shanghai, China), ligated into a Pucm-T vector (Sangon, Shanghai, China), and sequenced by Sanger’s method.

### 2.5. Sequence Analysis

The amino acid sequences of *T. dendrolimi* in the fasta format was used to query the sequence database of the National Center for Biotechnology Information (NCBI) to identify proteins with primary sequence similarity to *TdTPS* and *TdTRE*. Multiple sequence alignment was constructed using MEGA 7 [48] with the CLUSTAL V method [49,50]. Phylogenetic trees were constructed using the neighbor-joining (NJ) method [51]. *D. melanogaster* was used as the out-group, and the stability of the tree was assessed via bootstrapping with >1000 replicates.

### 2.6. Expression of TdTPS and TdTRE

Total RNA was extracted from 0.1 g of *T. dendrolimi* at larval, prepupal, pupal, and adult stages using a TransZol Up Plus RNA Kit (TransGen, Beijing, China). First-strand cDNA was synthesized from 1 μg total RNA using a FastQuant RT Kit With gDNase (Tiangen, Beijing, China).

Steps to construct linearized plasmid standards were as described previously with some modifications [52]. First, products *TdTPS* and *TdTRE* were extracted and purified with an agarose gel Extraction Kit (Sangon, Shanghai, China). Second, each gene was cloned separately using the Pucm-T Cloning Vector Kit (Sangon), according to the manufacturer instructions. Third, positive clones screened by PCR were processed for plasmid isolation using a Plasmid Extraction & Purification Kit (Sangon) and confirmed by Sanger sequencing (Sangon). Fourth, plasmids were completely linearized by *EcoR* I digestion for 4.5 h at 37 °C and confirmed by checking band patterns in the agarose gel. Fifth, linearized plasmids were quantified using a NanoDrop2000 spectrophotometer (NanoDrop), and copy numbers were calculated for all standards by the following formula [53]:

$$\mathrm{Copies}/\mu L\;=\;\frac{(6.02\;\times \;10^{23}\;\mathrm{copies})\;\times \;(\mathrm{plasmid}\;\mathrm{concentration}\;g/\mu L)}{\left(\mathrm{number}\;\mathrm{of}\;\mathrm{bases})\;\times \;(660\;\mathrm{daltons}/\mathrm{base}\right)}$$

Finally, the standard DNA (template) was prepared in a dilution series from 10^−3^ to 10^−10^ (copies/5 μL) for qPCR. qPCR was performed using a SYBR Green Mix Kit (Tiangen, Beijing, China) to measure the cycle number (Ct) of each dilution in duplicate. Each PCR reaction was mixed with 10 μL SYBR Green Mix, 6 μL dd H_2_O, 2 μL cDNA, and 1 μL of each primer (10 μM). Cycling conditions for all standards were described as above followed with dissociation curve analysis. Standard curves were generated as linear regression between Ct and log10 starting copy number of standard DNA. The Ct values were reported by the MX3000P MXPro program (Agilent Technologies, Palo Alto, USA). Amplification efficiency, slopes, and correlation coefficient (R^2^) were automatically calculated by the program.

To study the gene expression profiles during the four life stages, absolute quantitative PCR (AQ-PCR) was conducted to estimate their starting copy numbers. Gene specific primers, qTdTPSF/R, and qTdTREF/R, were designed according to the full-length cDNAs and these are listed in Table 1. RT-PCR was performed to obtain gene targets in the following cycling condition: initial denaturation 3 min at 95 °C followed by 40 cycles including 5 s at 95 °C, 10 s at 55 °C, 15 s at 72 °C. In the case of the *T. dendrolimi* samples, qPCR Ct values of *TdTPS* and *TdTRE* expression profiles were used to estimate starting copy numbers based on their standard curves.

### 2.7. Enzyme Activity Measurements

To obtain crude extracts for enzyme activity study, each sample (0.0100 ± 0.0002 g of larvae, pupae, prepupae, and adults) was homogenized at 0 °C (TGrinder OSE-Y20 Homogenizer, Tiangen, Beijing, China) after adding 2000 μL of 20 mM phosphate buffered saline (PBS, pH 5.8). The homogenates were centrifuged at 10,000× *g* at 4 °C for 20 min (CP100MX, Hitachi, Tokyo, Japan), and cuticle debris was removed. Supernatants in PBS were maintained at −80 °C to analyze TPS and TRE activity.

To determine TPS activity, the method of Dual et al. [54] was used. The qualitative analysis of trehalose was performed by thin layer chromatography (TLC); the quantitative analysis of trehalose synthase activity was investigated by examining the difference in glucose that was formed from the hydrolysis of maltose by TPS in the presence and absence of α-glucosidase and measured by DNS. The reaction mixture, containing 150 μL of crude extract and 100 μL substrate (10% maltose) was inactivated in a water bath at 60 °C for 1 h, and then in boiling water bath for 10 min. After adding 85% phosphoric acid to adjust pH to 4.2, 1 mL of diluted maltase (alpha-glucosidase) was added to the mixtures. The mixtures were inactivated in a water bath at 60 °C for 1 h and then in a boiling water bath for 10 min. Diluting and volumetrizing the reaction solution, adding 1:2 (V reaction solution: V DNS method), boiling water bath for 10 min, cooled in an ice bath, then the absorbance was measured at 550 nm.

TRE activity was measured as described previously by the 3,5-dinitrosalicylic acid colorimetric method (DNS method) with absorbance measured at 540 nm [55,56]. One unit (U) of enzyme activity was defined as the amount of enzyme capable of releasing 1 mg of reducing sugar per minute. The reaction mixture consisted of 500 μL crude extract and 500 μL DNS solution. The reaction was stopped by heating in boiling water for 5 min, then 4 mL of a pH 5.8 KH2PO4-NAOH buffer solution was added.

### 2.8. Statistical Analyses

Each treatment was performed using three biological and three technical replicates. Multifactor analysis (PROC GLM) of variance was conducted to evaluate temperatures and effect of developmental stage on the gene expression and enzyme activities of parasitoids using Tukey’s test. The qualities of biological parameters were compared using one-way analysis of variance (ANOVA) and multiple comparisons of means were conducted using Tukey’s test. Before analysis, percentage data were arcsine square root−transformed, and the data on the number of adults produced were log_10_-transformed to fit a normal distribution. For absolute quantification analysis, the number of molecules was expressed as the mean of the log_10_-converted value ± standard error. In all experiments, differences among means were considered significant at *p* < 0.05. Statistical analyses were conducted using SPSS 22.0 software (SPSS Inc., Chicago, IL, USA).

## 3. Results

### 3.1. Cloning and Characterization of Full-Length TdTPS and TdTRE cDNAs

**Figure 1:**
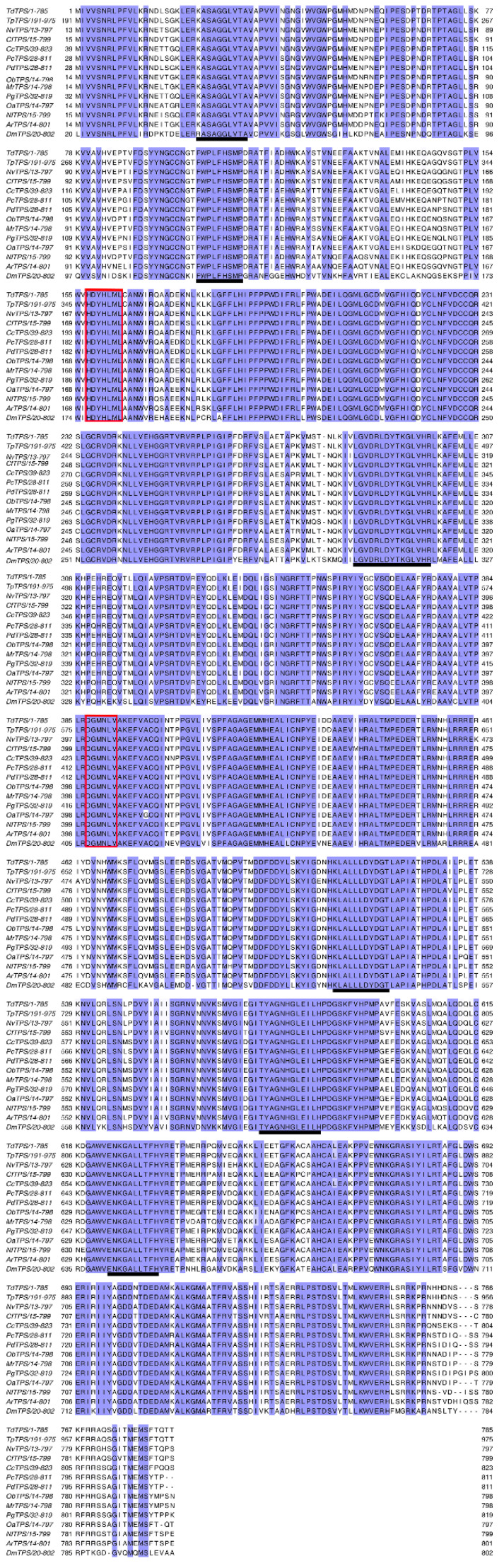
Alignment of *TdTPS*. Multiple alignment of trehalose-6-phosphate synthase (TPS) protein sequences from different insect species. Identical amino acid residues are shown in purple. The conserved motifs and the signatures are underlined and boxed, respectively.

**Figure 2:**
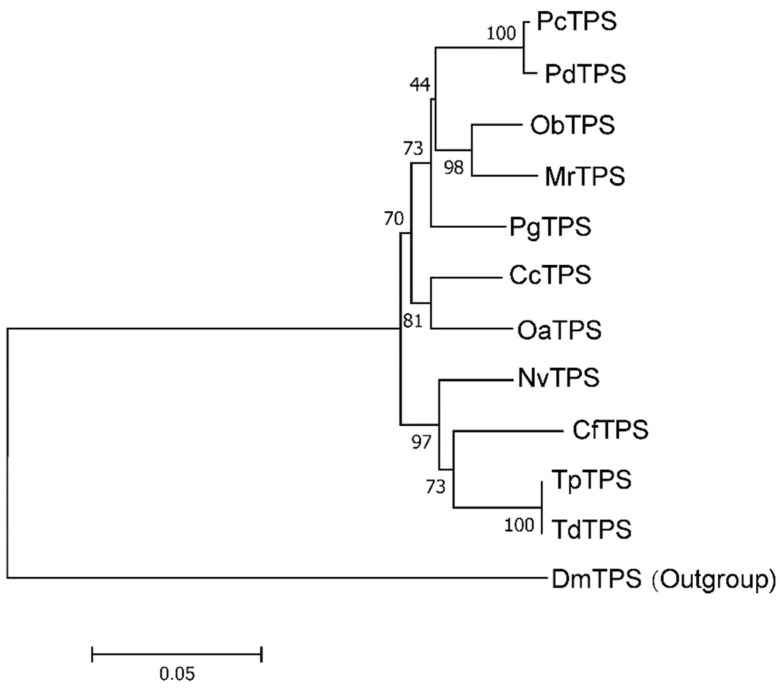
Phylogenetics of *TdTPS*. Phylogenetic tree constructed using the neighbor-joining (NJ) method. Percentage bootstrap values larger than 40 are shown on each branch. *PcTPS*: *Polistes canadensis*, XP_014609582; *PdTPS*: *Polistes dominula*, XP_015172546; *ObTPS*: *Osmia bicornis*, XP_029055554; *MrTPS*: *Megachile rotundata*, XP_003702415; *PgTPS*: *Pseudomyrmex gracilis*, XP_020289281; *CcTPS*: *Cephus cinctus*, XP_015588847; *OaTPS*: *Orussus abietinus*, XP_012281922; *NvTPS*: *Nasonia vitripennis*, XP_016837588; *CfTPS*: *Copidosoma floridanum*, XP_014213166; *TpTPS*: *Trichogramma pretiosum*, XP_014221069; *DmTPS*: *Drosophila melanogaster*, ABH06641.1.

To identify the *TdTPS* and *TdTRE*, cDNA fragments involved in trehalose metabolism were identified through *T. dendrolimi* transcriptome data (Hiseq 2000, Illumina, Santiago, USA). Based on the cDNA fragments, specific primers were performed and full-length cDNA of *TdTPS* and *TdTRE* was obtained by RACE-PCR. The full length *TdTPS* gene has 3189 bp, and the cDNA has a 2358 bp open reading frame (ORF), encoding a polypeptide of 785 amino acids with an estimated molecular weight of approximately 88.60 kDa and a pI of 6.56. The ORF was identical to the homolog from *Trichogramma pretiosum* (100%) (Table 2). Sequence analysis showed that the deduced amino acid sequence includes a conserved TPS domain (aa 1–478, E = 1 × 10^−^^133^^)^ and a TPP domain (aa 515–739, E = 7 × 10^−^^34^). Multiple protein alignment indicated that the *TdTPS* protein contains six conserved motifs (Figure 1). In addition to these specific motifs, *TdTPS* proteins contain the highly conserved domains, HDYHLML and DGMNLV, the same as TPS genes previously reported, such as in *D. melanogaster*, *Catantops pinguis,* and *Delia antiqua* [20,34,41]. Phylogenetic analysis showed that the *TdTPS* was more closely related to those of other Hymenoptera species (*T. pretiosum*, *Nasonia vitripennis,* and *Copidosoma floridanum*), and could be assigned to the same subgroup (Figure 2). The cloned TPS gene was designated as *TdTPS* and deposited into GenBank (MT108781).

**Figure 3:**
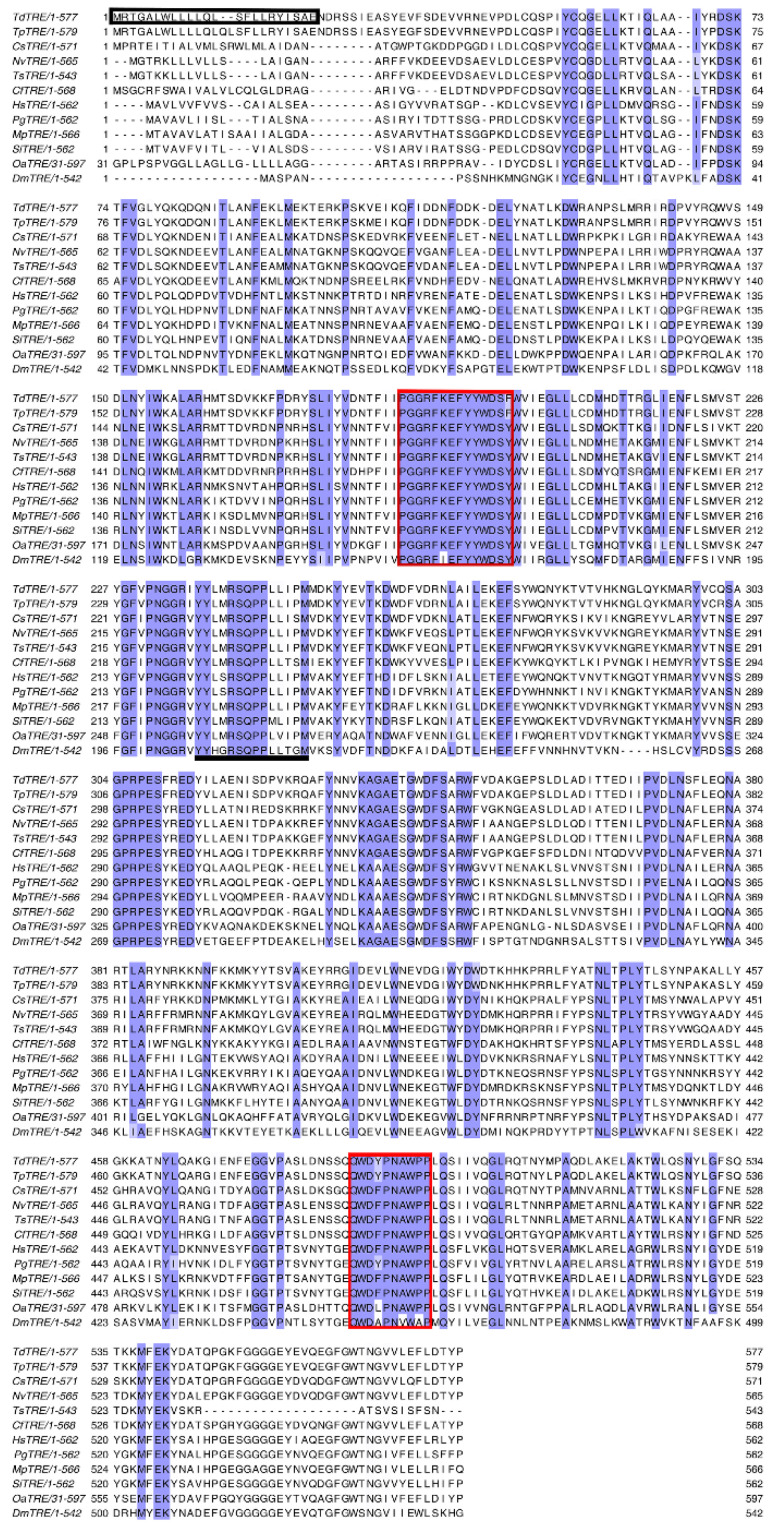
Alignment of *TdTRE*. Multiple alignment of soluble trehalase (TRE) protein sequences from different insect species. Identical amino acid residues are shown in purple. The conserved motifs, the signatures, and signal peptide sequence are underlined, red boxed, and black boxed, respectively.

**Figure 4:**
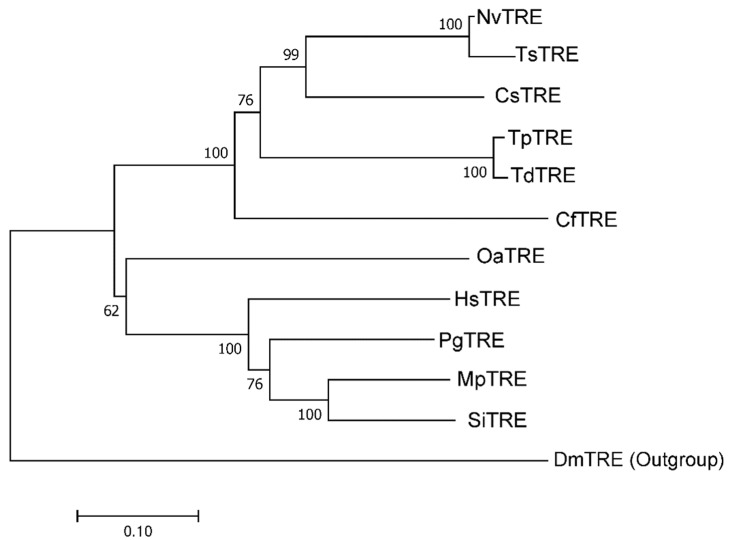
Phylogenetics of *TdTRE.* Phylogenetic tree constructed using the neighbor-joining (NJ) method. Percentage bootstrap values larger than 40 are shown on each branch. *NvTRE*: *Nasonia vitripennis*, XP_008215783; *TsTRE*: *Trichomalopsis sarcophagae*, OXU30694; *CsTRE*: *Ceratosolen solmsi marchali*, XP_011497766; *TpTRE*: *Trichogramma pretiosum*, XP_014236786; *CfTRE*: *Copidosoma floridanum*, XP_014216724; *OaTRE*: *Orussus abietinus*, XP_012271873; *HsTRE*: *Harpegnathos saltator*, XP_011144292; *PgTRE*: *Pseudomyrmex gracilis*, XP_020280302; *MpTRE*: *Monomorium pharaonis*, XP_028048276; *SiTRE*: *Solenopsis invicta*, XP_011170317; *DmTRE*: *Drosophila melanogaster*, NP_726025.1.

The full length *TdTRE* cDNA consisted of 2228 bp, including an 1878 bp open reading frame, encoding 625 amino acids with a predicted molecular weight of 73.2 kDa and a pI of 6.40. Basic Local Alignment Search Tool (BLAST) analysis revealed that *TdTRE* is 55.95–97.61% identical in structure to other known insect TRE forms. *TdTRE* is also most similar to the TRE from *T. pretiosum* (97.61%) (Table 2). The deduced amino acid sequence of *TdTRE* contains one conserved motif (YYLMRSQPPLLIPM) and a signal peptide sequence (Figure 3). Moreover, *TdTRE* had the same signature motifs, PGGRFREFYYWDSY and QWDYPNAWPP. For phylogenetic analysis, *TdTRE* was clustered with *T. pretiosum* TRE (Figure 4). Based on the sequence identity with known soluble form trehalase genes, *TdTRE* was identified as a soluble trehalase and deposited into GenBank (MT108782).

### 3.2. Standard Curve

**Figure 5:**
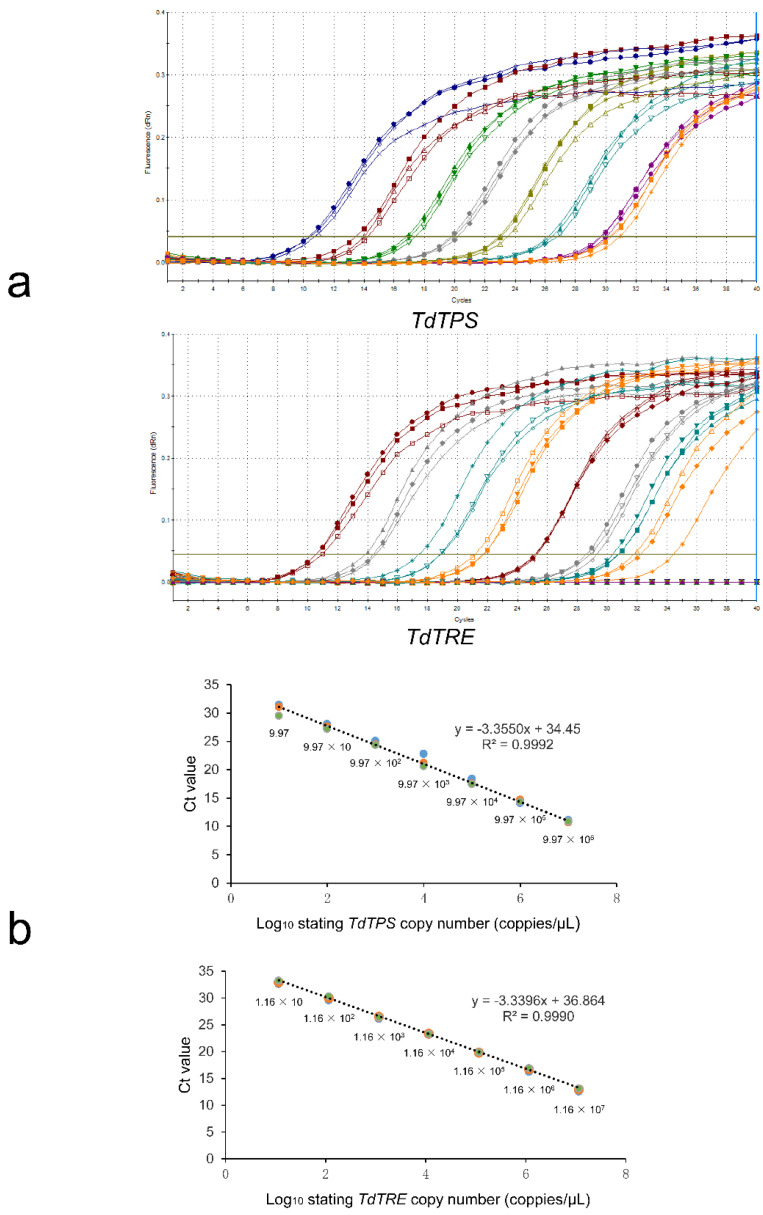
Standard curve for RT-qPCR amplification of standard sample. (**a**) Amplification plots for *TdTPS* and *TdTRE*; (**b**) standard curves of real-time PCR of *TdTPS* and *TdTRE*, using the method of absolute quantitative analysis, showing the testing in triplicate of a 10-fold dilution series containing a standard sample ranging from 9.97 × 10^6^ to 9.97 and 1.16 × 10^7^ to 1.16 × 10^1^ copies per.

Absolute quantification determines the actual copy numbers of target genes (*TdTPS* and *TdTRE*) by relating the Ct value to a standard curve and amplifying serial dilutions of plasmid standards by qPCR. The Ct values were measured and plotted against known copy numbers of the standard sample. The reaction efficiency and linearity for the serially diluted standards were of good quality for both genes (Figure 5). The standard curve covered a linear range of seven orders of magnitude. The slope (−3.3550 and −3.3396) and the correlation coefficient (R^2^ = 0.9992 and 0.9990) of the standard curve indicated that this assay could be used to quantify target RNA in *T. dendrolimi*.

### 3.3. Effect of Temperature on the Expression of TdTPS and TdTRE during Development

Absolute quantification PCR (AQ-PCR) experiments to measure *TdTPS* and *TdTRE* absolute expression in four developmental stages at different temperatures revealed that there was interaction between two factors on the mRNA levels of the genes (Table 3). Table 4 shows that the levels of the two genes were more highly expressed at the optimum storage temperature (13 °C) in all developmental stages compared to the expression at the other treatment temperatures. The *TdTRE* transcripts were highly expressed at 16 °C. In contrast, at the optimum development temperature (27 °C), the expression levels of *TdTPS* and *TdTRE* were low in all developmental stages. *TdTPS* was highly expressed in prepupae when *T. dendrolimi* developed at the three treatment temperatures, and *TdTPS* expression level was also higher in adults when they developed at 16 °C. For trehalase, the absolute expression levels of *TdTRE* were very low in the larval stage at all temperatures, and much higher in the prepupal stage at 27 °C and 16 °C.

### 3.4. Changes in Enzyme Activities

*TdTPS* and *TdTRE* enzyme activities were compared at different temperatures and different developmental stages, respectively. Comparison among temperatures indicated that *TdTPS* activities were similar at normal and cold temperatures. In larval and adult stages, *TdTRE* showed much higher activities at 16 °C and 13 °C. However, the *TdTRE* activity in prepupae showed the opposite result. In the pupal stage, *TdTRE* activities were similar at the three temperatures (Table 3 and Table 5).

At 27 °C, *TdTPS* activity in the developmental stages was similar. *TdTRE* had significantly higher activity in the prepupal and pupal stages and then declined in the adult stage. The enzyme activity of *TdTPS* and *TdTRE* had a similar trend at 16 °C and 13 °C in all *T. dendrolimi* developmental stages. Enzyme activities of *TdTRE* in prepupae declined sharply when they developed at 13 °C and 16° C.

### 3.5. In Vitro Rearing at Different Temperatures

The developmental durations and biological parameters at the five temperatures are shown in Table 6. The development of *T. dendrolimi* at 13 °C, 16 °C, 20 °C, and 23 °C prolonged for 21 days, 17 days, 8 days, and 5 days, respectively compared with those reared at the optimum temperature (27 °C). There were no significant differences in pupation rate, emergence rate, female proportion and number of normal adults among 16 °C, 20 °C, 23 °C, and 27 °C (*F*_4,14_ = 4.070, 135.396, 2.430, and 64.434, *p* = 0.033, 0.000, 0.116 and 0.000, respectively). The biological parameters (except female proportion) of *T. dendrolimi* reared on artificial medium were significantly affected by temperature. These parameters at 13 °C was lowest compared to that of other test temperatures.

## 4. Discussion

In this study, only the TPS gene of *T. dendrolimi* was obtained. The deduced amino acid sequence reveals that *TdTPS*, similar to the *DaTPS* gene in *Delia antiqua* [20], has two conserved functional domains that include an N-terminal TPS domain and a C-terminal TPP domain [43]. This result supports the conclusion that the insect TPS is a fused gene [57]. There are two signature motifs (HDYHL and DGMNLV) of TPS protein sequences for insects, plants, bacteria, fungi, and nematodes. Multiple protein alignment results show that, besides the signature motifs, *TdTPS* has other conserved motifs. Two types of trehalase exist in insects, a soluble trehalase and a membrane-bound trehalase with a transmembrane domain near the C-terminus [2,29,44,58,59]. Based on the conserved motif and specific signatures in deduced amino acid sequences, we identified the existence of the soluble form of trehalase (*TdTRE*) in in vitro reared *T. dendrolimi*.

Trehalose occurs in all insect species and is the most characteristic sugar in the insect hemolymph [57]. However, it has not been detected in the developmental stages of some species [2,23]. In the present study, *TdTPS* and *TdTRE* were expressed in larval, prepupal, pupal, and adult stages, whether they developed at the optimum development temperature or at sustained low temperatures. The results suggest that sustained low temperature has a strong effect on the expression level of trehalase genes in *T. dendrolimi*, which may facilitate the utilization of trehalose. A comparison of the expression levels of *TdTPS* and *TdTRE* at the tested temperature showed that sustained low temperature can upregulate their expression, but the expression of *TdTRE* was much lower than *TdTPS*. These indicated that the anabolism of trehalose was greater than its catabolism during low temperature development. Meanwhile, the reduction of trehalase activity inhibits the trehalose catabolism, which indirectly helps the trehalose accumulation. *TdTPS* might play a more important role than *TdTRE* in cold induction.

Prepupal stage is a critical developmental period for *Trichogramma*. The nutrient (host egg liquid) has been consumed by the end of larval stage and digestion of this food allows for the accumulation of energy used by the following pupal and adult stages. There were studies that have reported that the prepupa is the best stage for short-term storage of *Trichogramma* spp. reared in vivo [12]. In a previous study, sustained temperatures at 13 °C for 4 weeks was the optimum short-term storage condition for prepupae of in vitro reared *T. dendrolimi* [12]. Here, we explain the results of the previous study in terms of molecular physiology. *TdTPS* had a higher expression level in prepupae at the three test temperatures. Meanwhile, *TdTPS* had a high expression level in all stages when *T. dendrolimi* developed at low temperatures, especially at 13 °C. The enzymatic activity of *TdTPS* showed a similar change during the entire development. With a decrease of temperature, the activity increased. *TdTRE* showed a high expression level, but the enzymatic activity decreased at low temperatures in the prepupal stage. This trend was opposite to that observed at 27 °C. These findings indicate that the gene regulation for *TdTRE*, the soluble trehalase, might not determine the enzyme activity directly. However, there is no evidence to prove whether other soluble trehalase or membrane-bound trehalase genes exist in *T. dendrolimi* yet. Large-scale expression of TPS by insects before pupation promotes the synthesis of trehalose, leading to a high level of trehalose in the pupa stage. Under sustained low temperature, trehalase activity was inhibited in the prepupae since trehalose accumulation was probably required during this period to meet the energy required for chitin synthesis in the pupa and for adult emergence, suggesting its potential role in molting from pre-pupae to pupae. However, *TdTRE* is more active than *TdTPS* during cold stress. In vitro reared *T. dendrolimi* does not diapause under sustained low temperature stress, but cold only prolongs the development period after which development proceeds normally. In addition, the trehalose supplemented in artificial medium [9] needs to be consumed. On the other hand, in vitro reared *T. dendrolimi* could complete development above 13 °C because the trehalose added to the artificial medium may improve its cold tolerance. This protected the parasitoids, especially the young larvae, from low temperature injury. Trehalose was synthesized and accumulated at the same time for metabolism and utilization during the period. This indicates that trehalose might regulate growth of in vitro reared *T. dendrolimi* and the metabolic process of cold tolerance.

Trehalose concentration in insect blood hemolymph is not under homeostatic regulation. It is based on environmental conditions, physiological state, and nutrition. *Trichogramma* complete their development in the host egg before adult emergence. During development, the accumulation of trehalose helps the insects to resist environmental temperature stress and the stress of host malnutrition. A comparison of the trehalose contents and trehalase activity of *T. dendrolimi* produced in vitro and in vivo showed that the adults produced in vitro had higher trehalose content and trehalase activity over 10 generations [11]. Therefore, the responses and resistance of egg parasitoids to environmental stress may be different from those of other insects.

Future work will need to focus on the effects of different hosts (nutrition) on trehalose metabolism in *T. dendrolimi*, and determine how the trehalase genes are regulated under stress conditions.

## 5. Conclusions

Trehalose synthetase and soluble trehalase genes were identified from *T. dendrolimi* reared on an artificial medium. Sustained low temperature stress had different effects on trehalose metabolism related enzyme genes and enzyme activities of *Trichogramma*. The anabolism and catabolism of trehalose maintained a dynamic balance in the process of metabolism. Trehalose indeed accumulated as an energy source to be used in adverse conditions. *TdTPS* and *TdTRE* may be considered as an energy source and responsive enzymes for cold resistance in *Trichogramma*. The prepupa stage is a key period of *Trichogramma* development, in which the expression of genes involved in trehalose metabolism and corresponding enzyme activities undergo substantial changes. It’ is found that 13 °C appears to be the cold tolerance threshold temperature for in vitro reared *T. dendrolimi*. Since it remains unknown whether or not the in vitro reared *T. dendrolimi* could diapause without negatively affecting their reproductive parameters, we suggest that in vitro reared *T. dendrolimi* could be reared at temperatures of 16 °C, 20 °C, and 23 °C to reduce rearing costs.

## Figures and Tables

**Table 1:** Primers sequences used for real-time PCR.

Gene	Primer Name	Sequence (5′-3′)	Usage
*TdTPS*	5Race-R	TTTTTTTTTTTTTTTTTTTTTTTTTVN	
	UPM-long	CTAATACGACTCACTATAGGGCAAGCAGTGGTATCAACGCAGAGT	
	UPM-short	CTAATACGACTCACTATAGGGC	
	BD SMART II™ A Oligonucleotide	AAGCAGTGGTATCAACGCAGAGTACGCGGG	First full length cDNA synthesis for 5′cDNA amplification
	3’NUP-R	AAGCAGTGGTATCAACGCAGAGT	
	3Race-R	AAGCAGTGGTATCAACGCAGAGTACT(30)VN	First full length cDNA synthesis for 3′cDNA or CDS amplification
	5r-TPS-1R	AGTGGTCCGCGATGAAGGTCGC	
	5r-TPS-2R	CCCAGATGCCATTTCCATTGATGAC	5′ cDNA amplification
	cTPS-F	TCGGGCAGYATGATYGTCGT	
	cTPS-R	TGCCTCTCKACCCAYTTGAGCAT	
	cTPS-1R	TGTCTGTTACGTGGTCTGGGTGA	cds amplification
	3r-TPS-R	CCACCACGACAACTCCTCGA	3′ cDNA amplification
	qTPS-F	AATGGAAATGGCATCTGGGTC	
	qTPS-R	AGCAGCCGTTGTAGTACGAGTC	qRT-PCR
*TdTRE*	5r-TRE-1R	GTACAGCTCGTCCTTGTCATCG	
	5r-TRE-2R	CGCTAATTGTATCGTCTTTAGTAGTTCG	5′ cDNA amplification
	cTRE-F	TTCCTGAAACAGTAGTMTTTAGTCG	
	cTRE-R	CTCAAAAGTACGTTGTCCAAATAGAT	cds amplification
	3r-TRE-R	GTTGATATCAAGAAACCAACGAACG	3′ cDNA amplification
	qTRE-F	AAGCGAAAGCCAAGCAAGGT	
	qTRE-R	TGATACACGGGGTCACGAATAC	qRT-PCR

UPM, Universal Primer Mix; NUP, Nested Universal Primer; F, Forward; R, Reverse.

**Table 2:** The identities of *TdTPS* and *TdTRE* genes from different insects with *T. dendrolimi.*

Genes	Insects	GenBank Number	Identity
*TdTPS*	*Trichogramma pretiosum*	XP_014221069	100%
	*Nasonia vitripennis*	XP_016837588	94.78%
	*Copidosoma floridanum*	XP_014213166	93.89%
	*Cephus cinctus*	XP_015588847	92.87%
	*Osmia bicornis*	XP_029055554	92.45%,
	*Megachile rotundata*	XP_003702415	92.32%
	*Polistes canadensis*	XP_014609582	92.21%
	*Polistes dominula*	XP_015172546	92.08%
	*Orussus abietinus*	XP_012281922	91.96%
	*Pseudomyrmex gracilis*	XP_020289281	91.85%
	*Neodiprion lecontei*	XP_015522281	91.30%
	*Athalia rosae*	XP_012252443	90.83%
*TdTRE*	*Trichogramma pretiosum*	XP_014236786	97.61%
	*Trichomalopsis sarcophagae*	OXU30694	67.52%
	*Ceratosolen solmsi marchali*	XP_011497766	67.18%
	*Nasonia vitripennis*	XP_008215783	65.94%
	*Copidosoma floridanum*	XP_014216724	60.41%
	*Harpegnathos saltator*	XP_011144292	57.38%
	*Solenopsis invicta*	XP_011170317	56.69%
	*Monomorium pharaonic*	XP_028048276	56.42%
	*Pseudomyrmex gracilis*	XP_020280302	56.31%
	*Orussus abietinus*	XP_012271873	55.95%

**Table 3:** Multifactor variance analysis of effects of two factors on gene absolute expression and enzymes activity of *Trichogramma dendrolimi* reared on artificial medium.

Parameters	Factors	df	*F*	*p*
Absolute expression of *TdTPS*	A	2	2348.95	<0.001
	B	3	107.13	<0.001
	A × B	6	29.89	<0.001
Absolute expression of *TdTRE*	A	2	462.30	<0.001
	B	3	337.41	<0.001
	A × B	6	8.25	<0.001
Activity of *TdTPS*	A	2	25.24	<0.001
	B	3	18.12	<0.001
	A × B	6	1.41	0.252
Activity of *TdTRE*	A	2	7.10	<0.001
	B	3	39.86	<0.001
	A × B	6	46.64	<0.001

A, temperature; B, developmental stage.

**Table 4:** The gene expression of *TdTPS* and *TdTRE* of in vitro-reared *Trichogramma dendrolimi* reared at different temperatures and developmental stages.

Gene	Developmental Stage	Gene Absolute Expression (Copy Number (Coppies/μL))		
		13 °C	16 °C	27 °C
*TdTPS*	Larva	46175.06 ± 6494.615 A c	1419.43 ± 109.256 B b	1082.30 ± 53.480 B b
	Prepupa	221227.25 ± 8108.441 A a	9516.15 ± 473.110 B a	1809.35 ± 72.933 B a
	Pupa	84851.80 ± 5186.980 A b	4581.10 ± 1153.243 B b	859.34 ± 20.148 B bc
	Adult	66806.48 ± 1349.493 A bc	9716.46 ± 799.299 B a	774.88 ± 37.565 C c
*TdTRE*	Larva	8.36 ± 0.933 A c	9.72 ± 0.758 A b	2.41 ± 0.566 B c
	Prepupa	126.08 ± 7.207 A b	158.51 ± 10.949 A a	17.78 ± 1.506 B a
	Pupa	122.12 ± 4.192 A b	136.25 ± 7.018 A a	11.48 ± 2.241 B ab
	Adult	162.04 ± 12.807 A a	129.57 ± 3.250 A a	11.19 ± 0.886 B b

Mean ± SE were calculated from three replicates. Mean ± SE followed by the same capital letter within a row were not significantly different (Tukey’s test: *p* > 0.05); Mean ± SE followed by the same lowercase letter within a column were not significantly different (Tukey’s test: *p* > 0.05).

**Table 5:** Activity of the enzymes involved in trehalose metabolism of in vitro-reared *Trichogramma dendrolimi* reared at different temperatures and developmental stages.

Enzymes	Developmental Stage	Activity [μg/mL (Extract/min)]		
		13 °C	16 °C	27 °C
*Td* *TPS*	Larva	0.15 ± 0.009 A ab	0.14 ± 0.012 A b	0.09 ± 0.014 A a
	Prepupa	0.12 ± 0.005 A b	0.11 ± 0.009 A b	0.08 ± 0.021 A a
	Pupa	0.12 ± 0.015 A b	0.11 ± 0.006 A b	0.09 ± 0.006 A a
	Adult	0.20 ± 0.014 A a	0.19 ± 0.008 A a	0.11 ± 0.010 B a
*Td* *TRE*	Larva	0.52 ± 0.032 A a	0.44 ± 0.028 A a	0.28 ± 0.022 B b
	Prepupa	0.22 ± 0.032 B b	0.18 ± 0.016 B b	0.49 ± 0.021 A a
	Pupa	0.47 ± 0.019 A a	0.50 ± 0.031 A a	0.50 ± 0.012 A a
	Adult	0.50 ± 0.013 A a	0.50 ± 0.002 A a	0.22 ± 0.012 B b

Mean ± SE were calculated from three replicates. Mean ± SE followed by the same capital letter within a row were not significantly different (Tukey’s test: *p* > 0.05); Mean ± SE followed by the same lowercase letter within a column were not significantly different (Tukey’s test: *p* > 0.05).

**Table 6:** Developmental quality of in vitro reared *T. dendrolimi* at different temperatures.

Temperature	Developmental Duration (D)					Biological Parameters			
	Egg	Larva	Prepupa	Pupa	Total Duration	Pupation Rate (%)	Emergence Rate (%)	Female Proportion (%)	Number of Normal Adults
27 °C	2	2	2	4	10	96.77 ± 0.391 a	77.44 ± 1.451 a	88.16 ± 0.555 a	876.00 ± 48.03 a
23 °C	3	3	2	5	13	96.44 ± 0.441 ab	75.16 ± 1.326 a	87.50 ± 0.620 a	805.33 ± 35.044 a
20 °C	4	4	3	7	18	96.48 ± 0.395 ab	76.59 ± 0.728 a	87.62 ± 0.949 a	814.67 ± 13.119 a
16 °C	5	9	4	9	27	94.88 ± 1.35 ab	75.86 ± 2.499 a	88.00 ± 0.392 a	904.00 ±72.746 a
13 °C	6	9	7	9	31	92.81 ± 1.041 b	36.03 ± 1.159 b	85.55 ± 0.724 a	92.67 ± 5.897 b

Mean ± SE values were calculated from three replicates. Mean ± SE followed by the same lower case letter within a column are not significantly different (Tukey’s test: *p* *<* 0.05).
